# Association Between Metformin and Abdominal Aortic Aneurysm: A Meta-Analysis

**DOI:** 10.3389/fcvm.2022.908747

**Published:** 2022-05-23

**Authors:** Wenqiang Niu, Juan Shao, Benxiang Yu, Guolong Liu, Ran Wang, Hengyang Dong, Haijie Che, Lubin Li

**Affiliations:** ^1^Department of Vascular Surgery, Yantai Yuhuangding Hospital, Yantai, China; ^2^Department of Dermatology, Yantai Yuhuangding Hospital, Yantai, China; ^3^Nursing Department, Heze Medical College, Heze, China

**Keywords:** metformin, abdominal aortic aneurysm, expansion, mortality, meta-analysis

## Abstract

**Objective:**

To systematically examine the association between metformin and abdominal aortic aneurysm (AAA) and provide a basis for the treatment of AAA.

**Methods:**

Pubmed, Embase, Cochrane Library, and Ovid databases were searched by computer to identify the literature related to metformin and AAA published until February 2022. The literature was screened according to the inclusion and exclusion criteria, data were extracted, and a quality assessment was conducted. The meta-analysis was performed using Stata 16.0 and RevMan 5.3 software.

**Results:**

Seven articles containing a total of 10 cohort studies (85,050 patients) met the inclusion criteria and were included in the review. Meta-analysis showed that metformin can limit the expansion of AAA (MD = – 0.72, 95% CI: – 1.08 ~ −0.37, *P* < 0.00001), as well as reduce AAA repair or AAA rupture-related mortality (OR = 0.80, 95% CI:0.66 ~ 0.96, *P* = 0.02). The difference was statistically significant (*P* < 0.05).

**Conclusion:**

Metformin can limit the expansion of AAA and reduce the incidence of AAA and postoperative mortality. However, further biological experiments and clinical trials still need to be conducted to support this.

## Introduction

Abdominal aortic aneurysm (AAA) is a common asymptomatic disease with a prevalence of 1.2–4% in people over the age of 50 (1–4). If left untreated, AAA can progress to aneurysm rupture and seriously threaten the patient's life. Despite significant advances in research on the genetic, metabolic, and environmental risks associated with aortic aneurysms and population-based disease screening programs, thousands of patients die prematurely due to aneurysm-related events. Studies have reported that the average annual mortality rate from aortic aneurysms worldwide is 2.8/100,000, which has increased by 12% over the past 20 years (5).

AAA remains a significant preventable cause of death in the early twenty-first century. According to the guidelines, aggressive surgical intervention is recommended for larger diameter AAA or symptomatic AAA. However, with improved screening and appropriate examination equipment, many cases of AAA can be detected at a very young age when they are asymptomatic. However, regular imaging can only monitor this type of AAA. Relevant studies have reported that up to 70% of this type of AAA will eventually grow to the size requiring surgical repair (6, 7). Currently, there is no medical treatment that can inhibit the growth of small AAA. Therefore, in recent years, domestic and international researchers have been committed to exploring the non-surgical treatment of AAA, hoping to limit the further expansion of AAA in a non-invasive fashion and achieve the goal of avoiding surgical treatment or delaying the time of operation.

Despite the fact that numerous clinical trials have proven that pharmacological interventions are ineffective in limiting the AAA enlargement or disease progression, studies in various population groups have demonstrated a negative correlation between diabetes and the prevalence, growth, and rupture of AAA (8–10). The causal mechanism behind this correlation is not yet precisely understood but may be related to hyperglycemia, the effect of antidiabetic drugs, or other factors of the diabetic environment (11, 12). It is well-known that metformin is one of the most commonly used drugs for the treatment of diabetes. Experimental studies have shown that it inhibits the proliferation of aortic smooth muscle cells and the expression of extracellular matrix metalloproteinase (MMP)-2. It also reduces oxidative stress (13–16). Therefore, metformin could potentially become a drug that will be used in the future to treat AAA.

Nevertheless, the association between metformin and AAA remains unclear. To help clarify the available epidemiological evidence, we conducted a meta-analysis of the literature on metformin and AAA published until February 2022 to further explore the relationship between metformin and AAA.

## Methods

### Search Strategy

This meta-analysis was performed in accordance with the Preferred Reporting Items for Systematic Reviews and Meta-Analyses (PRISMA) (17, 18). The literature on metformin and AAA was systematically searched in Pubmed, Embase, Cochrane Library, and Ovid databases until February 2022. The search terms included “Metformin (Dimethylbiguanidine, Dimethylguanylguanidine, Glucophage, Metformin Hydrochloride, Hydrochloride, Metformin, Metformin HCl, HCl, Metformin),” “Aortic aneurysm (Aneurysms, Aortic, Aortic Aneurysms, Aneurysm, Aortic),” “Aortic dilatation,” and “Cohort Study.” Two reviewers (RW and HD) independently browsed all articles and selected only cohort studies to be included in this review. There were no language restrictions for the included studies. The analysis was based on published articles and conference abstracts with available statistical data and charts. Authors were contacted to provide additional data from their studies, if necessary. The relevant literature from the reference lists of the included articles was also manually searched, screened, and reviewed.

### Criteria for Inclusion and Exclusion

In our opinion, if these studies were cohort studies in adult diabetic patients with a minimum duration of 8 weeks of metformin pharmacologic intervention, the article clearly demonstrates the effect of metformin pharmacologic therapy on change in aortic aneurysm diameter or on events related to aortic dilation as outcome results, we consider these studies eligible for inclusion. The cohort study articles contained risk estimates of OR, RR, or HR with a corresponding 95% CI.

We excluded (1) observational and retrospective studies; and (2) Studies with Intervention Duration <8 Weeks, and (3) reviews, case reports, comments, recommendations, letters, ongoing trials, protocols, conference abstracts, consensus or statements, and articles lacking applicable data; and (4) studies that could not be utilized, such as duplicate reports, as well as studies of poor quality, of an inconsistent type, or with too little information, and (5) studies with inappropriate statistical methods and insufficient data.

### Data Extraction

Two researchers (RW and HD) searched for relevant articles, screened eligible articles based on the inclusion and exclusion criteria, and independently extracted data using a standardized datasheet. Disagreements were resolved by discussion with a third researcher (GL). We extracted basic information (author, year, country), type of study, inclusion criteria, participants and their number, interventions (metformin), and results. We compared treatment strategies for aortic changes with or without metformin medication in patients with aortic aneurysms, without the limitation of treatment history. The results of evaluation were as follows: trends in aortic diameter and AAA repair or AAA rupture related mortality.

### Quality Assessment

The Newcastle-Ottawa Scale (NOS) was used to evaluate the quality of all studies (19). The NOS checklist contains three quality parameters: (1) selected population, (2) comparability, and (3) assessment of exposure or outcome in cohort studies. Each study was scored from zero to nine. Studies that scored ≥ seven were considered high-quality articles.

### Data Analysis

Statistical analyses were performed using Stata16.0 and Review Manager 5.3 software. We used MD and the corresponding 95% CI to account for continuous data and OR to account for dichotomous results (20). *P* <0.05 indicated statistical significance. The I^*2*^ statistic, which reflects the proportion of heterogeneity, was used to analyze inconsistency in the results. With I^2^ <50% and insignificant heterogeneity, the fixed-effects model was used. With I^2^ ≥ 50% and significant heterogeneity, the sensitivity analysis was performed to identify the source of heterogeneity (21). The random-effects model was used if the cause of heterogeneity could not be determined (22).

## Results

### Study Inclusion

**Figure 1:**
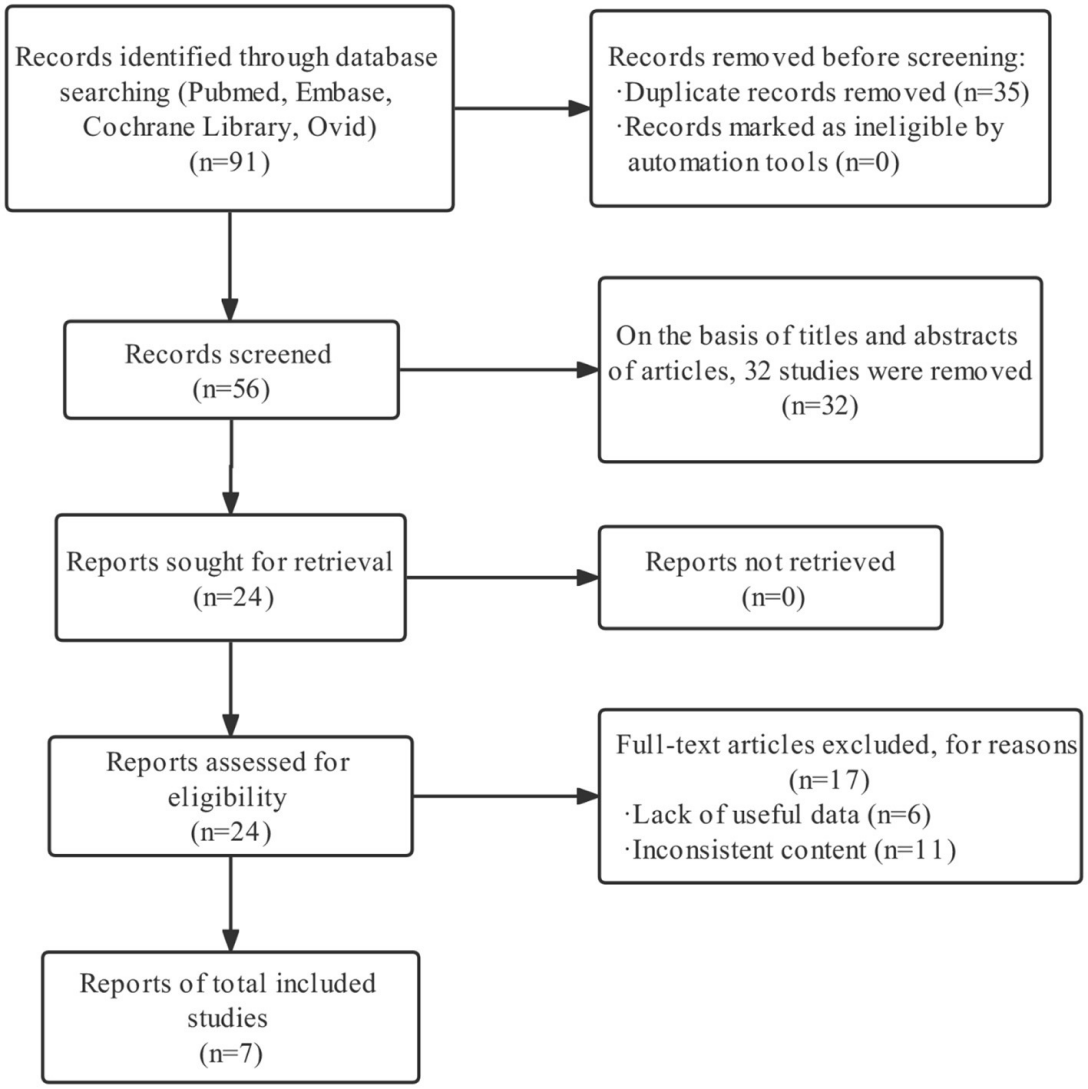
Flow chart presenting the screening process.

By searching the relevant literature, 91 articles were retrieved and screened according to the established inclusion and exclusion criteria, and a total of seven articles were included in the review (Figure 1). The seven articles contained a total of 10 cohort studies with 85,050 participants. The relevant characteristics of the included literature are displayed in Table 1.

**Table 1:** Characteristics of the included studies.

**Study authors and country**	**Publication year**	**Study design**	**Participants**	**Follow-up duration (mean years)**	**Outcomes**	**Adjusted OR (95% CI)/ rates of enlargement (mm/year)**	**Quality scores**
Fujimura et al. (23), America	(2016)	Cohort studies	Elderly patients with diabetes with untreated AAA (*n* = 58)	2.6	Maximum aortic diameter growth measured by contrast-enhanced CT	• Patients with AAA prescribed metformin, 0.4 ± 0.6 (*n* = 15) • Patients with AAA not prescribed metformin, 1.7 ± 0.5 (*n* = 43)	7
Golledge et al. (24), Australia and New Zealand	(2017)	Cohort studies 1	Elderly patients with AAA (infrarenal aortic diameter ≥ 30 mm) (*n* = 1,357)	3.6	Maximum aortic diameter growth measured by ultrasound	• Patients with AAA prescribed metformin, 1.03 ± 2.68 (*n* = 118) • Patients with AAA not prescribed metformin, 1.62 ± 2.45 (*n* = 1,239)	• 8
Golledge et al. (24), Australia and New Zealand	(2017)	Cohort studies 2	Elderly patients with AAA (infrarenal aortic diameter ≥ 30 mm) (*n* = 287)	2.9	Maximum aortic diameter growth measured by contrast enhanced CT	• Patients with AAA prescribed metformin, 1.40 ± 2.99 (*n* = 39) • Patients with AAA not prescribed metformin,2.55 ± 3.04 (*n* = 248)	8
Golledge et al. (24), Australia and New Zealand	(2017)	Cohort studies 3	Elderly patients with AAA (infrarenal aortic diameter ≥ 30 mm) (*n* = 53)	1	Maximum aortic diameter growth measured by Philips Medical Systems	Patients with AAA prescribed metformin, 0.37 ± 1.28 (*n* = 16) Patients with AAA not prescribed metformin, 1.46 ± 1.52 (*n* = 37)	8
Itoga et al. (25), America	(2018)	Cohort studies	Elderly patients with diabetes and a diagnosis of AAA without rupture (*n* = 13,843)	4.2	Maximum aortic diameters growth determined from radiographic reports	Patients with AAA prescribed metformin, 1.2 ± 1.9 (*n* = 5,496) Patients with AAA not prescribed metformin, 1.5 ± 2.2 (*n* = 8,347)	8
Golledge et al. (26), Australia	(2019)	Cohort studies 1	Patients with asymptomatic unrepaired AAA of any diameter ≥ 30 mm (*n* = 1,080)	3.2	The combined incidence of AAA repair (open or endovascular) or mortality due to AAA rupture (defined as AAA events)	• Patients with AAA prescribed metformin, 0.63 ± 1.42 (*n* = 129) • Patients with AAA not prescribed metformin,1.15 ± 1.99 (*n* = 105)	8
Golledge et al. (26), Australia	(2019)	Cohort studies 2	Patients with asymptomatic unrepaired AAA of any diameter ≥50 mm (*n* = 763)	3.6	The combined incidence of AAA repair (open or endovascular) or mortality due to AAA rupture (defined as AAA events)	• Patients with AAA prescribed metformin, 0.48 ± 1.58 (*n* = 106) • Patients with AAA not prescribed metformin, 1.16 ± 2.72 (*n* = 75)	8
Unosson et al. (27), Sweden	(2021)	Cohort studies	Patients with initial abdominal aortic diameter ≥30 mm (*n* = 98)	3.2	Maximum aortic diameter growth measured by ultrasound	• Patients with AAA prescribed metformin, 1.1 ± 1.1 (*n* = 65) • Patients with AAA not prescribed metformin, 1.6 ± 1.4 (*n* = 33)	8
Sutton et al. (28), American	(2020)	Cohort studies	Male patients with AAA (*n* = 67,434)	5	Surgery and/or death after the diagnosis of AAA	• Death 0.88 (0.85 to 0.91) • Death after surgery 0.93 (0.84 to 1.04)	8
Turowicz et al. (29), Poland	(2021)	Cohort studies	Patients undergoing AAA repair (*n* = 77)	0.1	Maximum aortic diameter growth measured by contrast enhanced CT	0.09 (0.02 to 0.47)	7

### Study Quality

According to the NOS scale, the quality evaluation scores of seven cohort study articles were ≥ 7, and the quality was high (Table 2).

**Table 2:** The Newcastle-Ottawa Scale (NOS).

**Study authors**	**Quality evaluation**	**Representativeness of the exposed cohort (1)**	**Selection of the non-exposed cohort (1)**	**Ascertainment of exposure (1)**	**Demonstration that the outcome of interest was not present at the start of the study (1)**	**Comparison of the ability of cohorts based on the design or analysis (2)**	**Assessment of the outcome (1)**	**Was follow-up long enough for the outcomes to occur (1)**	**Adequacy of the cohort follow-up (1)**
Fujimura et al. (23)	7	1	1	1	1	1	0	1	1
Golledge et al. (24)	8	1	1	1	1	2	1	0	1
Itoga et al. (25)	8	1	1	1	1	1	1	1	1
Golledge et al. (26)	8	1	1	1	1	1	1	1	1
Unosson et al. (27)	8	1	1	1	1	1	1	1	1
Sutton et al. (28)	8	1	1	1	1	1	1	1	1
Turowicz et al. (29)	7	1	1	1	1	1	1	0	1

### Result of the Meta-Analysis

#### Association Between Metformin and Enlargement of Abdominal Aortic Diameter

**Figure 2:**
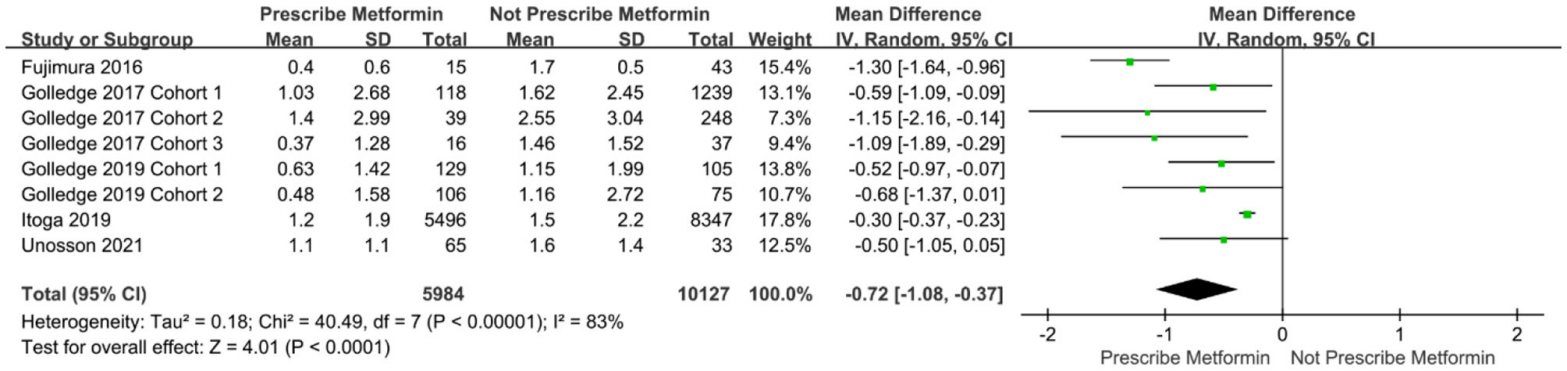
Forest plot for the enlargement of abdominal aortic diameter.

We analyzed eight cohort studies that enrolled 16,111 patients (prescribe metformin: 5,984 participants; not prescribe metformin: 10,127 participants) to assess the association between metformin and abdominal aortic diameter enlargement. The analysis showed significant heterogeneity among the studies (I^2^ = 83%, *P* <0.00001). Forest plots showed that metformin inhibited the enlargement of abdominal aortic diameter compared to patients not treated with metformin drugs (MD = −0.72, 95% CI: – 1.08 ~ −0.37, *P* <0.00001) (Figure 2).

**Figure 3:**
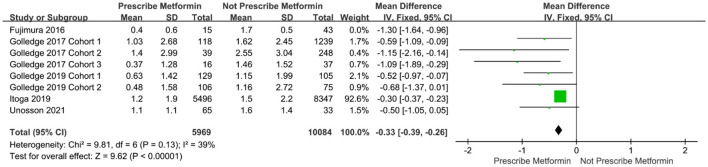
Forest plot after excluding the data on the enlargement of abdominal aortic diameter by Fujimura et al.

The Egger test was subsequently performed. The results showed *t* = −3.03 (*P* = 0.023), indicating that the meta-analysis findings were subject to publication bias (30, 31). To identify the source of heterogeneity, we further performed the sensitivity analysis and re-did the meta-analysis, removing studies using the item-by-item method. After excluding Fujimura et al., the meta-analysis showed significantly reduced heterogeneity among the studies (I^2^ = 39%, *P* <0.00001) (Figure 3).

#### AAA Repair or AAA Rupture-Related Mortality

**Figure 4:**
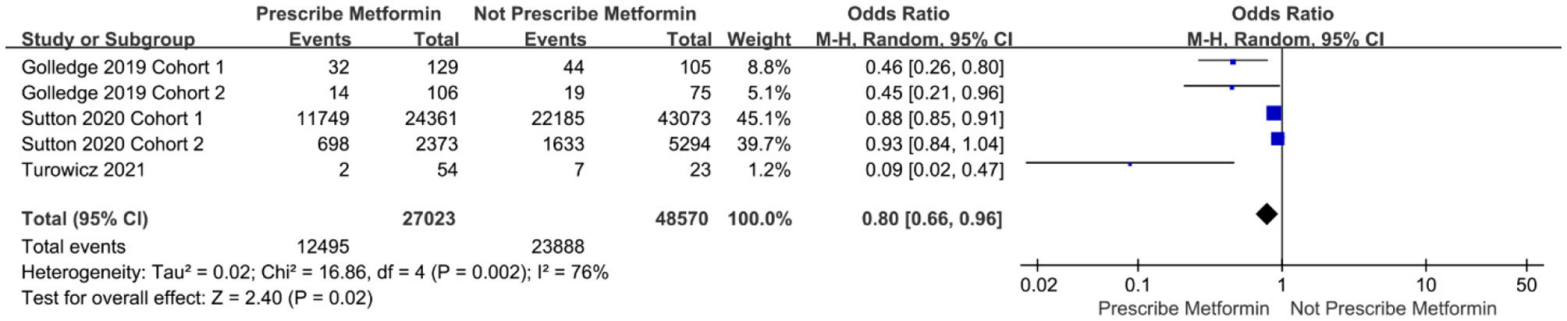
Forest plot for AAA repair or AAA rupture-related mortality.

Five cohort studies with 75,593 patients analyzed AAA repair or AAA rupture-related mortality changes. There was significant heterogeneity among the cohort studies (I^2^ = 76%, *P* = 0.002). The results showed that AAA repair or AAA rupture-related mortality was significantly lower in the group taking metformin than in the group not taking metformin (OR = 0.80, 95%*CI*: 0.66 ~ 0.96, *P* = 0.02). Further, the Egger test showed *t* = −2.12 (*P* = 0.124) (Figure 4).

## Discussion

In this study, a meta-analysis of seven included cohort studies was conducted, in which the relationship between metformin and AAA was examined using the degree of abdominal aortic diameter enlargement as an index. The results showed that metformin limited the enlargement of AAA diameter. At the same time, metformin also reduced AAA repair or AAA rupture-related mortality.

Over the past 20 years, significant progress has been made in the open or endovascular surgical treatment of large, symptomatic, and ruptured AAA. However, there is still a gap in the pharmacological inhibition of minor AAA enlargement. Although numerous clinical trials have shown that pharmacological interventions are ineffective in limiting AAA expansion or disease progression, the available evidence suggests that patients with diabetes are less likely to develop AAA. When an aneurysm is present, they also have a slower rate of aneurysm progression or expansion. Despite the many common risk factors for the development of aneurysmal and cardiovascular diseases, the paradoxical relationship between diabetes and aneurysmal disease remains controversial and insufficiently substantiated for interpretation (32, 33).

In one of the largest studies on the association between diabetes and AAA, which involved 5,697 patients, the results showed that AAA increased by an average of 2.2 mm per year. However, patients with diabetes had an average decrease in AAA growth of 0.5 mm per year compared to patients without diabetes (34). A study by Kristensen et al. (35) reported that AAA patients with high hemoglobin A1c had a slower rate of increase in the aortic vessel diameter than patients with low hemoglobin A1c, regardless of whether the patients had diabetes. A prolonged hyperglycemic environment leads to glycosylation of the extracellular matrix, which further leads to cross-linking of collagen and elastin in the aortic wall. Compared to non-cross-linked collagen, the cross-linked extracellular matrix is more difficult for proteases to cleave, which reduces the release of matrix metalloproteinases and cytokines from inflammatory cells such as monocytes (8, 36). However, a study conducted in 2017 involving 628,264 people who were screened for vascular disease reported that among people without diabetes, the risk of developing an aortic aneurysm increased with higher blood glucose levels in the non-diabetic range, while patients diagnosed with diabetes had a lower risk of aortic aneurysm (37). From this report, we can suggest that the negative association between diabetes and the prevalence and growth of an aortic aneurysm may be due to treatment rather than the presence of diabetes.

In the pharmacological treatment of diabetes, metformin is the drug used for the longest treatment. In cardiovascular disease, there is increasing evidence that metformin protects blood vessels by inhibiting the production of reactive oxygen species, the activity of inflammatory nuclear factor-kB, targets of the mammalian rapamycin (antifungal antibiotic) pathway, autophagy, and mural angiogenesis (38). In rodent studies, it has been reported that metformin inhibits pathological mechanisms of aortic inflammation, extracellular matrix remodeling, and oxidative stress involved in AAA (23, 39).

Metformin is one of the most commonly used drugs for the treatment of diabetes. To support our results, researchers have conducted animal experiments with metformin and modeled an AAA model with porcine pancreatic elastase in normoglycemic mice. The results showed that the incidence and growth rate of AAA were significantly decreased in mice taking metformin. At the same time, it was also shown that in the AAA mouse model modeled by angiotensin II, metformin had a protective effect on aneurysms, and this protective mechanism was associated with its ability to activate the AMP-activated protein kinase (AMPK) pathway (39). In addition, as oral hypoglycemic drugs, thiazolidinediones (e.g., pyrazine, rosiglitazone, etc.) regulate peroxisome proliferator-activated receptor-γ (a nuclear hormone receptor family transcription factor), thereby affecting the activity of matrix metalloprotein−9 (MMP-9) and the release of cytokines. Dipeptidyl peptidase—4 (DPP-4) inhibitors (e.g., salbutamol, sitagliptin, etc.) can also reduce the production of reactive oxygen species (ROS) by mitochondria.

A study by Hsu et al. (40) on the relationship between the use of oral hypoglycemic drugs and the risk for aortic aneurysm showed that in patients with diabetes, that risk was lower in patients taking metformin than in those not taking it. Another study by Kristensen et al. (41) showed that patients who took metformin for a prolonged period of time had a lower rate of abdominal aortic aneurysm enlargement relative to those who took metformin for a long time. This also supports the finding that metformin limits the expansion of abdominal aortic aneurysms. Eight additional meta-analyses, including 29,587 patients, reported that metformin significantly inhibited aortic aneurysm enlargement in patients with abdominal aortic aneurysms (95% CI: −1.38 ~ −0.28) (42). A recent meta-analysis of 10 studies reported that in patients with type 2 diabetes, metformin drugs reduced the rate of aneurysm diameter growth (*MD* = −0.67, 95% CI: – 1.20 ~ −0.15) and the probability of AAA occurrence (OR = 0.61, 95% *CI*: 0.4 ~ 0.92) (43). This is similar to the results of our review, in which we included more experimental studies that were cohort-based, potentially reducing selection bias. The results indicated that metformin inhibits the progression of aortic aneurysm diameter and reduces the incidence of abdominal aortic aneurysm and associated mortality after abdominal aortic aneurysm surgery.

Relevant studies have shown that atherosclerosis and AAA share common risk factors, including gender, advanced age, hypertension, and smoking. Therefore, atherosclerosis may be considered a potential pathophysiological mechanism of AAA (44, 45). Vessels are constantly stimulated by hemodynamic perturbations, which lead to changes in vascular smooth muscle cells and promote the release of matrix remodeling enzymes, resulting in vascular structure remodeling. At the same time, due to atherosclerosis, vascular smooth muscle cells get destroyed and lose their function, which promotes the expansion of the intima and ultimately leads to localized medial thinning and rupture of the aortic wall. Sarajlić et al. (46) speculated that hereditary diseases might correlate with diabetes, limiting the progression of AAA. The authors noted that multiple effector kinases are involved in both the AAA and atherosclerotic pathways, which may only be affected by genetic mutations that disrupt the aneurysm pathway. Chen et al. (47) have shown that metformin drugs are feasible for treating cardiovascular disease due to their anti-atherosclerotic and lipid-lowering effects. Some studies suggest that the anti-atherosclerosis effect of metformin may be related to its own effect, further reducing carotid intima-media thickness (CIMT) (48). Another explanation is that metformin drugs are able to inhibit macrophage apoptosis to a certain extent, reduce lipid deposition, and block the process of vascular atherosclerosis development (49).

Metformin has an omnidirectional mode of action, and although it has been used in the treatment of diabetes for more than 60 years, its exact mechanism of action in therapy is still not fully understood (50). According to the findings of *in vitro* studies and animal models, metformin may inhibit the pathological progression of AAA through a variety of mechanisms, including limiting aortic inflammation and reducing extracellular matrix remodeling and oxidative stress (23, 51). It has also been shown that an aneurysm enlargement that metformin inhibits may be associated with decreased secretion of matrix metalloproteinases (MMPs) and interleukin-6 (IL-6) (8). Despite this, there is no uniform consensus regarding the mechanism by which metformin limits the AAA enlargement, which warrants further experimental studies in this area.

In the included literature, studies on the relationship between metformin and AAA were confounded by multiple factors, including age, race, region, and other drugs used to treat diabetes. This may have biased the results of our meta-analysis. In general, we concluded that metformin limited the expansion of AAA, however, there was large variability in the studies. Through the sensitivity analysis, we were able to identify that the source of heterogeneity was likely in the Fujimura et al. (23) study. In that study, the dilatation rate of the abdominal aorta was much higher than in other included studies, and the follow-up time was also longer. However, the quality score for that study was high, its content was sufficient, and the experimental design was reasonable. Based on these quality assessment criteria, there was no reason to exclude the study from the meta-analysis, and its results also show that metformin limits the expansion of AAA.

The limitation of this meta-analysis is that there are only a few existing studies on the relationship between metformin and aortic aneurysm, so the number of articles included in the review is small. At the same time, some studies had smaller sample sizes and shorter follow-up times. Larger samples and longer follow-ups are still needed for further long-term exploration of the association between metformin and AAA. In addition, there is no uniform clinical standard for measuring and reporting AAA, which contributes to differences in accuracy and leads to heterogeneity.

## Conclusions

In summary, we performed a meta-analysis on the association between metformin and AAA. We found that metformin limits the expansion of AAA to a certain extent and reduces the incidence of AAA and postoperative mortality. However, since the mechanism by which metformin inhibits AAA is not precise, further biological experiments and clinical trials are needed to fully elucidate its mode of action.

## Data Availability Statement

The original contributions presented in the study are included in the article/supplementary material, further inquiries can be directed to the corresponding authors.

## Author Contributions

LL and WN: conception and design. GL, RW, and HD: collection and assembly of data. WN, JS, and BY: data analysis and interpretation and manuscript writing. LL and HC: revision of manuscript. All authors contributed to the article and approved the submitted version.

## Conflict of Interest

The authors declare that the research was conducted in the absence of any commercial or financial relationships that could be construed as a potential conflict of interest.

## Publisher's Note

All claims expressed in this article are solely those of the authors and do not necessarily represent those of their affiliated organizations, or those of the publisher, the editors and the reviewers. Any product that may be evaluated in this article, or claim that may be made by its manufacturer, is not guaranteed or endorsed by the publisher.
